# A three-population wave-of-advance model for the European early Neolithic

**DOI:** 10.1371/journal.pone.0233184

**Published:** 2020-05-19

**Authors:** Kenichi Aoki

**Affiliations:** Organization for the Strategic Coordination of Research and Intellectual Properties, Meiji University, Nakano-ku, Tokyo, Japan; University College Dublin, IRELAND

## Abstract

Ancient DNA studies have shown that early farming spread through most of Europe by the range expansion of farmers of Anatolian origin rather than by the conversion to farming of the local hunter-gatherers, and have confirmed that these hunter-gatherers continued to coexist with the incoming farmers. In this short report, I extend a previous three-population wave-of-advance model to accommodate these new findings, and derive the conditions supportive of such a scenario in terms of the relative magnitudes of the parameters. The revised model predicts that the conversion rate must, not surprisingly, be low, but also that the hunter-gatherers must compete more strongly with the converted farmers than with the alien farmers. Moreover, competition with the hunter-gatherers diminishes the speed of the wave-of advance of the farmers. In addition, I briefly consider how the wave-of-advance approach may contribute to interpreting the results of archaeological studies using the summed probability distribution of radiocarbon dates.

## 1. Introduction

A longstanding question with regard to the spread of early farming in Europe is whether it occurred by the range expansion of farmers of Near Eastern origin or by the iterative conversion to farming of the local hunter-gatherers. Genetic studies of ancient DNA have produced strong evidence in favor of the former proposal. Thus, the broad pattern appears to be one in which farmers migrated in from Anatolia and their descendants colonized Europe with minimal en route interaction or admixture with the indigenes [1]. More specifically, a principal component analysis shows that the early Neolithic individuals from central and western Europe including Spain, and possibly also southern Scandinavia, cluster together with the Anatolian farmers [2–4]. Similarly, Valdiosera et al. [5] estimate the genetic contributions of western hunter-gatherers, early Anatolian farmers, and Yamnaya (a later incursion) by a three-way admixture analysis and show that early Neolithic individuals from the north and south of Spain have greater than 95% Anatolian ancestry, with a small remaining contribution from the western hunter-gatherers. Equally important is the observation that the resident hunter-gatherers continued to coexist with the incoming farmers [6–10].

The seminal theoretical studies on the spread of early farming in Europe by Ammerman and Cavalli-Sforza [11, 12] (see also [13, 14]) were judiciously equivocal as to the two possibilities. On the one hand, these authors invoked the wave-of-advance model [15] to explain the approximately constant rate of spread of early farming. On the other hand, they argued that the frequency gradients of classical genetic markers currently seen in the southeast to northwest direction were formed during this process, which is more consistent with the second theory, as it may imply a gradual dilution of the Near Eastern genetic contribution. Note that the one-population Fisher model is not equipped to predict the formation of ancestry gradients. The principal components study by Menozzi et al. [16] that appeared to support this latter claim has subsequently been questioned on statistical grounds [17], and Arenas et al. [18] have suggested that these gradients were formed during the Palaeolithic. It has also been shown that the spread of early farming “was not … regular across Europe … but proceeded in leaps” [19]. However, approximate rate constancy does hold [20], and the truth may lie somewhere in between [21].

The above perspective on the initial phase of the Neolithic transition in Europe—farming was spread by populations of Anatolian descent at an approximately constant rate—needs to be qualified by the recognition of inevitable regional variation (e.g. [9, 10, 21, 22]). Doubts have even been raised on the applicability of the wave-of-advance (reaction-diffusion) approach [23]. Hence, what follows and in particular the relevance of the predictions made in this paper are highly dependent on how these issues are perceived.

**Fig 1:**
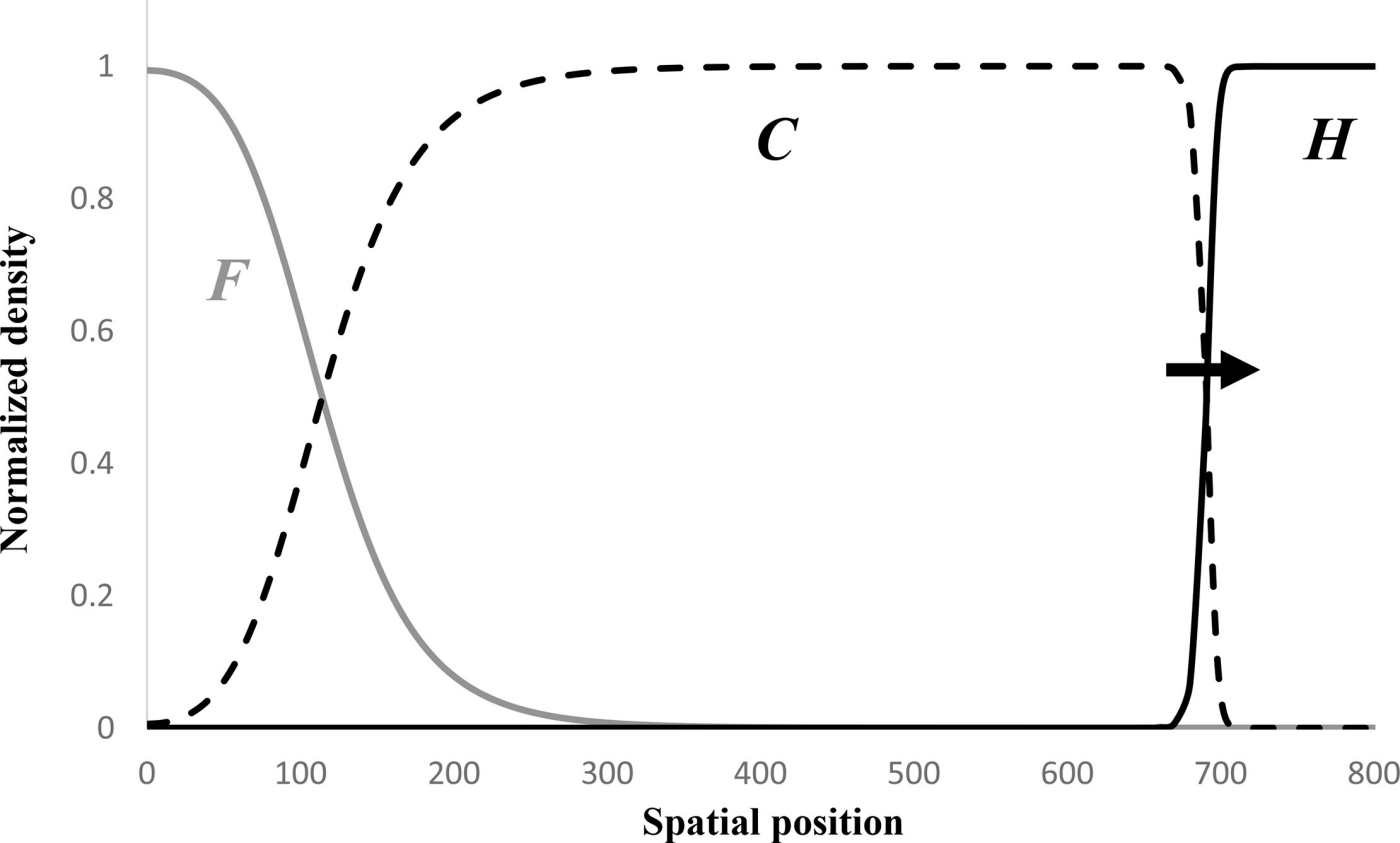
A numerically-simulated traveling wave solution in which farming spreads at a constant speed and an ancestry gradient is formed in its wake. Parameter values are *a* = 1, *b* = 1, *s* = 0.1, *g* = 2.1, *w* = *p* = *q* = 0. Solid grey, broken black, and solid black curves give the normalized densities of initial farmers *F*, converted farmers *C*, and hunter-gatherers *H*, respectively, on the interval 0≤*x*≤800. The black arrow marks the wave front.

Aoki et al. [24] proposed a three-population wave-of-advance model describing the joint dynamics of the incoming Near Eastern farmers (“initial” farmers), resident hunter-gatherers, and hunter-gatherers that have adopted farming (“converted” farmers). This model incorporated conversion of the hunter-gatherers to farming by horizontal cultural transmission and competition between the initial and converted farmers (“intra-subsistence” competition). Within this relatively simple setting, we identified four classes of traveling wave solutions. The solution we judged to be in qualitative agreement with the then accepted observations is reproduced in Fig 1. It shows a wave front of the converted farmers moving at constant speed and that, in its wake, reciprocal gradients form in the densities of the initial and converted farmers. None of the four classes of solutions predicted the steady range expansion of the initial farmers of Near Eastern descent that is coupled with the persistence of the hunter-gatherers, which is what is suggested by the ancient DNA data.

This model has subsequently been extended by Mimura and colleagues to allow for differential mobility in hunter-gatherers and farmers and then rigorously analyzed from a mathematical standpoint [25, 26]. Ackland et al. [27] have generalized the model by the addition of various interaction terms and a geographical barrier to dispersal; their numerical simulations suggest that the converted farmers may often mediate the spread of farming beyond the barrier.

The main purpose of the present paper is to revise the model of Aoki et al. [24] to be qualitatively consistent with our current understanding of the Neolithic transition in Europe and to address the limited question of the wave profile or composition of the traveling wave solutions, specifically whether the wave front of the spread of farming will be dominated by the initial farmers, and whether the hunter-gatherers will persist in the wake of the wave. More sophisticated models and analyses have been applied to the speed of the wave-of-advance [28, 29]. Finally, I suggest that the relevant traveling wave solution may provide a suitable null hypothesis for interpreting the results of archaeological studies using the so-called summed probability distribution of radiocarbon dates, e.g. [30, 31].

## 2. Revised model

Let *F*(*x*,*t*), *C*(*x*,*t*), and *H*(*x*,*t*) denote the densities of the initial farmers (Near Eastern descent), the converted farmers (European descent), and the hunter-gatherers (also European descent), respectively, at position *x* and time *t* in a linear habitat. The revised model is described by the equations

$$\begin{array}{l} F_{t}=DF_{xx}+r_{f}F[1-\left(F+C\right)/K-wH/L],\\ C_{t}=DC_{xx}+r_{c}C[1-\left(F+C\right)/K-pH/L]+e(F+C)H,\\ H_{t}=DH_{xx}+r_{h}H[1-H/L-q\left(F+C\right)/K]-e(F+C)H. \end{array}$$ (1)

(The subscripts *t* and *xx* indicate the time derivative and the second spatial derivative, respectively.) The original model [24] contained seven parameters: the diffusion constant *D*, assumed to be the same for all three populations; the intrinsic growth rates *r*_*f*_, *r*_*c*_, and *r*_*h*_ of the initial farmers, converted farmers, and hunter-gatherers; the carrying capacities *K* and *L* of the farmers and hunter-gatherers; and the conversion rate *e* of hunter-gatherers to farming. Here I add three terms, multiplied by parameters *w*, *p*, and *q*, which represent competition between the two types of farmers and the hunter-gatherers (“inter-subsistence” competition). Note the initial and converted farmers are assumed to have the same competitive effect, *q*, on the hunter-gatherers, and the intra-subsistence competition coefficients have been set to 1. Hence, the model is not completely general. All parameters in the revised model are positive.

It is convenient to work with the following non-dimensional equations

$$\begin{array}{l} F_{t}=F_{xx}+aF(1-F-C-wH)\\ C_{t}=C_{xx}+C(1-F-C-pH)+s(F+C)H\\ H_{t}=H_{xx}+bH[1-H-(q+g)(F+C)] \end{array}$$ (2)

where *F*, *C*, and *H* have been normalized by their carrying capacities; and *a* = *r*_*f*_/*r*_*c*_, *b* = *r*_*h*_/*r*_*c*_, *s* = *eL*/*r*_*c*_, *g* = *eK*/*r*_*h*_. The meanings of the symbols are summarized in Table 1.

**Table 1:** List of symbols.

Variables (except in Eq 1)
*x* rescaled position in linear space
*t* rescaled time
*F*(*x*, *t*) density of initial (alien) farmers normalized by carrying capacity, *K*
*C*(*x*, *t*) density of converted farmers normalized by carrying capacity, *K*
*H*(*x*, *t*) density of hunter-gatherers normalized by carrying capacity, *L*
Original parameters
*D* diffusion constant common to all individuals
*r*_*f*_ intrinsic growth rate of initial farmers
*r*_*c*_ intrinsic growth rate of converted farmers
*r*_*h*_ intrinsic growth rate of hunter-gatherers
*K* carrying capacity of initial and converted farmers combined
*L* carrying capacity of hunter-gatherers
*e* conversion rate of hunter-gatherers to farming
*w* competitive effect of hunter-gatherers on initial farmers
*p* competitive effect of hunter-gatherers on converted farmers
*q* competitive effect of farmers on hunter-gatherers
*v* minimum speed of wave-of-advance
Non-dimensional composite parameters
*a* = *r*_*f*_/*r*_*c*_
*b* = *r*_*h*_/*r*_*c*_
*s* = *eL*/*r*_*c*_
*g* = *eK*/*r*_*h*_

Numerical solutions of Eq 2 are computed on the finite interval 0≤*x*≤*l* with reflecting boundary conditions and from the initial conditions

F(x,0)=1;C(x,0)=0;H(x,0)=0for0≤x≤d, (3A)

F(x,0)=0;C(x,0)=0;H(x,0)=1ford<x≤l. (3B)

Eq 3B entails that before the spread of early farming European hunter-gatherers were at carrying capacity (unity in the non-dimensional variables). An anthropological study based on the ages at death of skeletons in Mesolithic cemeteries [32] suggests that this was approximately the case; farmers far behind the wave front were likely also at carrying capacity (also unity in the non-dimensional variables). Typically, I take *l* = 800 and *d* = 80. (The code written in an advanced form of BASIC is available upon request.)

## 3. Ad hoc analysis

The non-dimensional equations that ignore spatial structure, Eq A1, and their equilibria are given in Appendix 1. The traveling wave solutions of interest are those that connect a stable equilibrium E5 where $\hat{C}<<\hat{F}$ to equilibrium E2, as such solutions imply a wave front dominated by the initial farmers and persistence of the hunter-gatherers in its wake. Note

$$E2=(\hat{F}=0,\hat{C}=0,\hat{H}=1),$$ (4)

and

$$E5=\left(\hat{F}=\frac{\left(1-w)(p-w-s\right)}{\left(p-w)[1-w(q+g)\right]},\hat{C}=\frac{s(1-w)}{\left(p-w)[1-w(q+g)\right]},\hat{H}=\frac{1-q-g}{1-w(q+g)}\right).$$ (5)

A monostable wave connects a stable equilibrium to an unstable one. It is shown in Appendix 2 that E5 exists and that the necessary conditions for linear stability are met if

p>w+s,w<1,q+g<1. (6)

**Fig 2:**
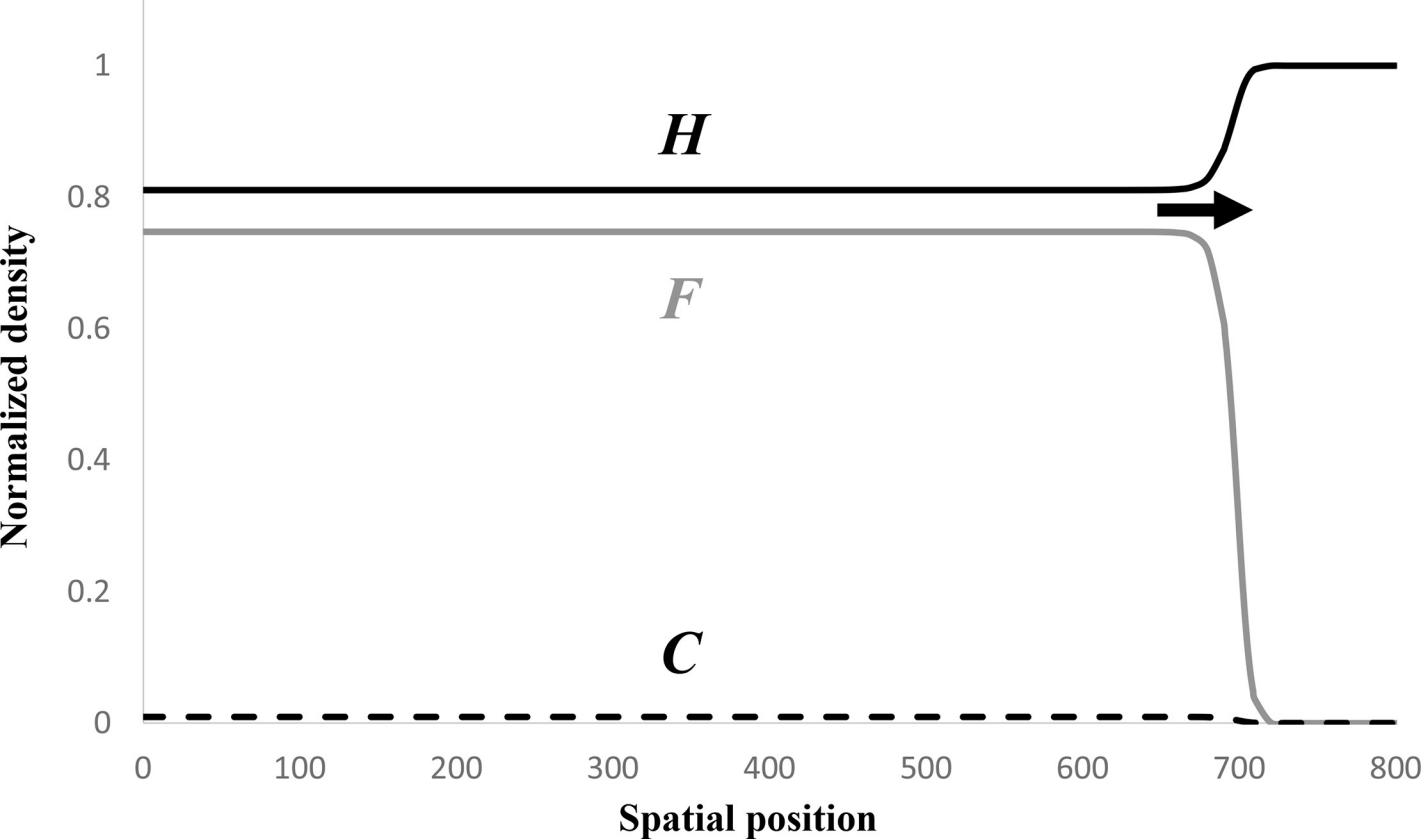
An example of a numerically-simulated monostable wave connecting E5 to E2 that is predicted for parameter range *p*>*w*+*s*, *w*<1, *q*+*g*<1. Specifically, *a* = 1, *b* = 1, *s* = 0.01, *g* = 0.15, *w* = 0.3, *p* = 1.1, *q* = 0.1. In particular, the values of *s* and *g* entail that the carrying capacity of farmers is 15 times that of hunter-gatherers [33]. Solid grey, broken black, and solid black curves give the normalized densities of initial farmers *F*, converted farmers *C*, and hunter-gatherers *H*, respectively, on the interval 0≤*x*≤800. It is worth noting that *F* and *H* are both below carrying capacity behind the wave front. The black arrow marks the wave front. The numerically estimated speed is 1.62, which is in good agreement with the heuristic minimum speed $v=2\sqrt{a(1-w)}$ = 1.67 (Appendix 3).

These conditions can be rewritten in terms of the original parameters by noting *s* =*eL*/*r*_*c*_ and *g* = *eK*/*r*_*h*_. Fig 2 illustrates a monostable wave generated under these conditions. I have been unable to obtain the sufficient conditions for linear stability of E5.

A bistable wave connects two stable equilibria. Appendix 2 shows that E5 and E2 cannot coexist as stable equilibria. Hence, there are no bistable waves that connect E5 and E2.

**Fig 3:**
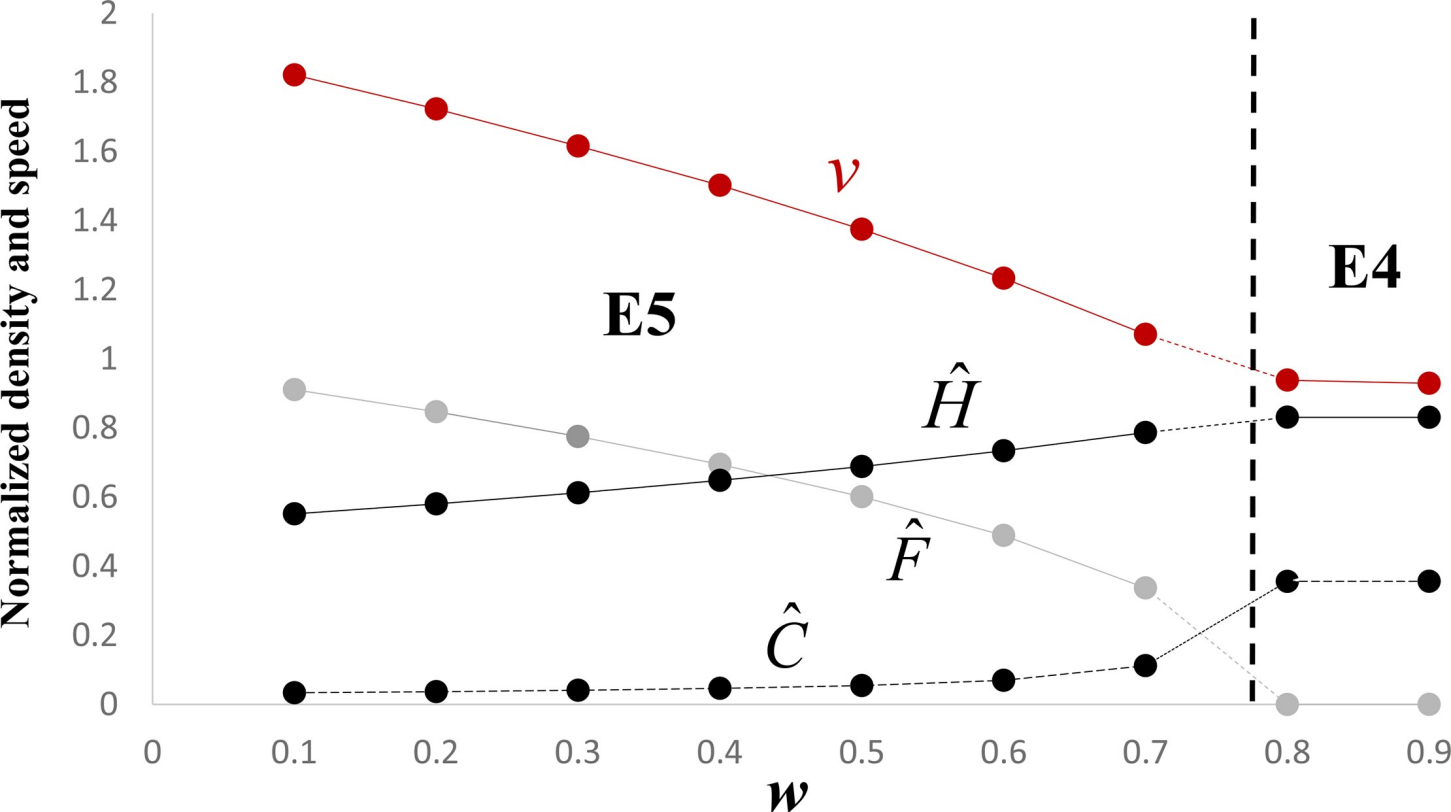
Dependence on *w* of the stable equilibrium behind the wave front. Fixed parameter values are *a* = 1, *b* = 1, *s* = 0.025, *g* = 0.375, *p* = 0.8, *q* = 0.1. Grey dots give the numerically-simulated normalized values of $\hat{F}$ for each value of *w*. Similarly, black dots connected by broken and solid lines give the values of $\hat{C}$ and $\hat{H}$, respectively. The brown dots give the numerically obtained speeds. Note the relevant equilibrium behind the wave front is E5 when *w* < *p* − *s* = 0.775 and E4 when the inequality is reversed. Similarly, the heuristically predicted speed is $v=2\sqrt{a(1-w)}$ when *w* < *p* − *s* = 0.775 and $v=2\sqrt{1+s-p}$ when the inequality is reversed (Appendix 3). For E5, $\hat{F}$ is monotone decreasing in *w*, $\hat{C}$ is monotone increasing in *w* because *p* = 0.8 < 1, and $\hat{H}$ is monotone increasing in *w*.

In terms of the original parameters, the revised model predicts a minimum speed of either

$$v=2\sqrt{Dr_{f}(1-w)},$$ (7A)

or

$$v=2\sqrt{D[r_{c}(1-p)+eL]},$$ (7B)

whichever is larger, for a monostable wave connecting E5 to E2, and incidentally also for a monostable wave connecting E4 to E2 (see Fig 3 and Appendix 3). These minimum speeds are slower than those proposed by Aoki et al. [24], because of the presence of the competition terms *w* and *p*. In particular, in the special case of *r*_*f*_≥*r*_*c*_(*a*≥1), the speed of a monostable wave connecting E5 to E2 is necessarily given by Eq 7A, which entails that the speed, as well as the density (see Eq 5 and Appendix 2), of the invading farmers is monotone decreasing in the competition coefficient *w*. On the other hand, when Eq 7B applies, the speed is monotone increasing in the conversion rate, *e*, in agreement with Fort [29].

**Fig 4:**
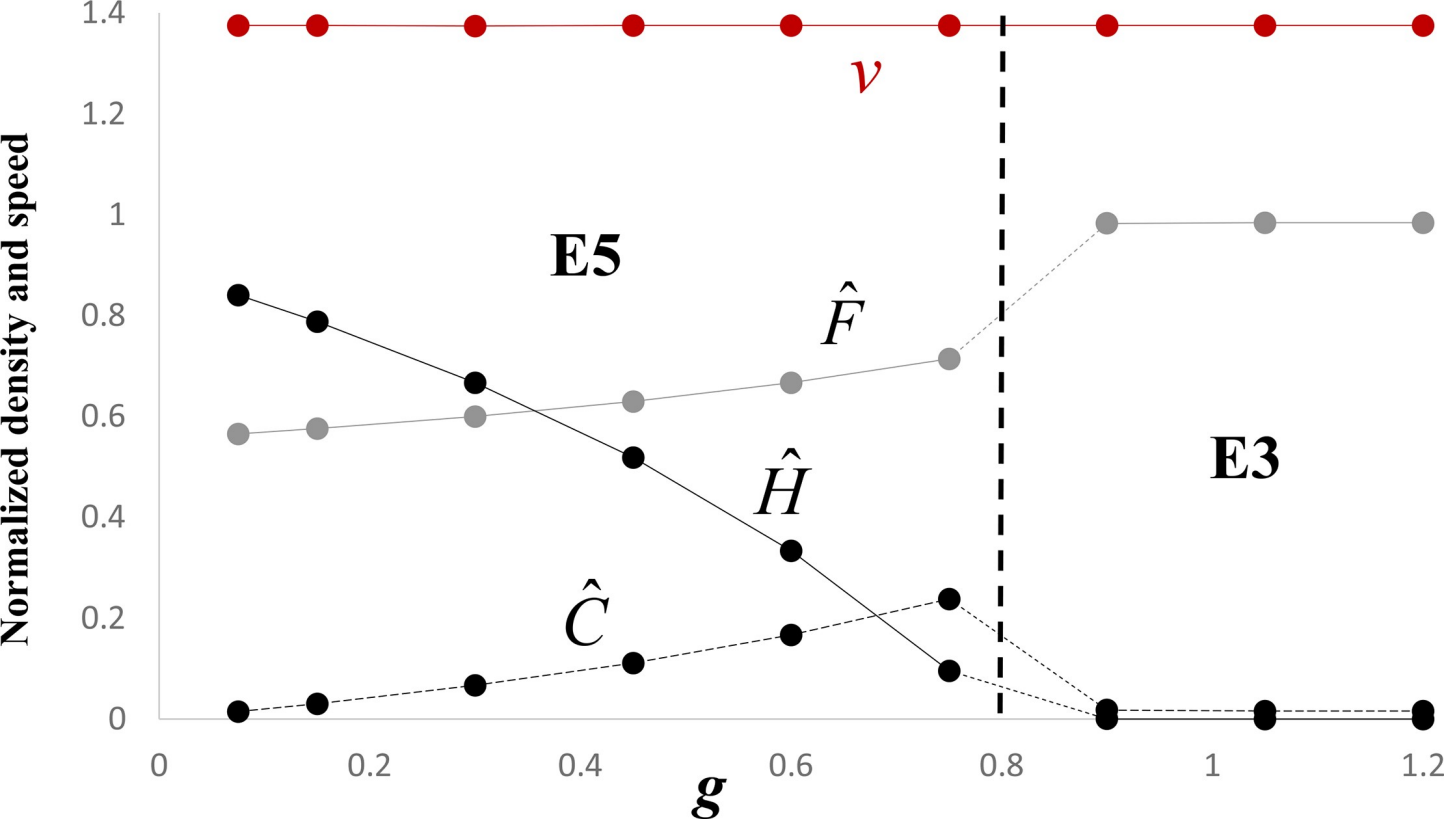
Dependence on *g* of the stable equilibrium behind the wave front, where *s* = *ξ* × *g* is assumed to covary with *g*. Fixed parameter values are *a* = 1, *b* = 1, w = 0.5, *p* = 0.7, *q* = 0.2, and *ξ* = 1/15. Grey dots give the numerically-simulated normalized values of $\hat{F}$ for each value of *w*. Similarly, black dots connected by broken and solid lines give the values of $\hat{C}$ and $\hat{H}$, respectively. The brown dots give the numerically obtained speeds. Note the relevant equilibrium behind the wave front is E5 when *g* < 1 − *q* = 0.8 and E3 when the inequality is reversed. The heuristically predicted speed is always $v=2\sqrt{a(1-w)}$, because 1 + *s* − *p* < *a*(1 − *w*) for the range of values of *s* (Appendix 3). For E5, $\hat{F}$ is monotone increasing in *g* because *ξ*<*w*(*p*−*w*)/(1−*wq*) = 1/9, $\hat{C}$ is monotone increasing in *g*, and $\hat{H}$ is monotone decreasing in *g*.

Figs 3 and 4 summarize the results of some additional numerical simulations, which are consistent with Eqs 6 and 7 and the analyses in Appendices 1–3. With regard to the speed, these figures show that the numerically obtained speeds closely approximate the heuristically predicted minimum speeds (Eqs 7A and 7B). Moreover, Fig 3 shows that the speed may decrease as a result of an increase in the competition coefficient *w*, whereas Fig 4 shows that an increase of conversion rate (*g* = *eK*/*r*_*h*_) does not necessarily entail an increase in speed.

## 4. Discussion

Recent genetic studies of ancient DNA have shown that early farming spread through Europe principally by the demic expansion of farmers of Anatolian origin rather than by cultural diffusion. They have also confirmed that the indigenous hunter-gatherers were not replaced, but continued to coexist for a while with the incoming farmers. In this paper, I extend the three-population wave-of-advance model of Aoki et al. [24] to accommodate these new findings. The three populations are the farmers of Anatolian descent (initial farmers, *F*), the farmers of European descent that have converted from hunting and gathering (converted farmers, *C*), and the hunter-gatherers that have maintained their lifestyles (*H*). I formulate the conditions for the scenario suggested by the ancient DNA studies to be realized, in terms of the competition coefficients between the three populations and the non-dimensional conversion rate of hunter-gatherers to farming, which are the relevant parameters of the model.

Depending on the parameter values, various traveling wave solutions are possible. Among these, the solution that is qualitatively most compatible with the ancient DNA observations is the one that connects the stable equilibrium E5 (Eq 5) to the unstable equilibrium E2 (Eq 4). Provided certain conditions are met, this solution entails that the wave front will be dominated by the initial farmers and that the hunter-gatherers will persist behind the wave front (Fig 2). The inequalities listed in Eq 6 indicate that such a solution is more likely to exist when the conversion rate (the normalized parameters *s* = *eL*/*r*_*c*_ and *g* = *eK*/*r*_*h*_ are proportional to the conversion rate *e*) is relatively low, the competitive effect of hunter-gatherers on the initial farmers (*w*) and of the farmers on the hunter-gatherers (*q*) are relatively low, and the competitive effect of hunter-gatherers on the converted farmers (*p*) is relatively high. In particular, *p* > *w* + *s* must be satisfied (Eq 6). When this inequality is reversed, the traveling wave solution may connect equilibrium E4 to equilibrium E2, which entails that the spread of farming is mediated by the converted farmers (see Appendix 1 and Fig 3).

Compatibility with the ancient DNA data also requires that the density of converted farmers behind the wave front be relatively low, which is predicted for small values of *s*, *g*, *w*, and *q*, and large values of *p* (Appendix 2). From a mathematical standpoint, these conditions are all intuitively reasonable. It is an interesting open question whether the hunter-gatherers (*H*) would have behaved less competitively toward the phenotypically divergent initial farmers (*F*) [34, 35] than toward the phylogenetically closer converted farmers (*C*); i.e. *w* < *p*. Conversion is modeled here as an instantaneous process, but may actually have occurred gradually [36, 37]. Populations in transition may have encroached on the resources used by the pristine hunter-gatherers, eliciting opposition from the latter.

In order that the model described in this paper apply to the Neolithic transition in Europe, the conversion rate of hunter-gatherers to farming must be low. An ethnological study shows that hunter-gatherers may have profitably coexisted with farmers, for example by trading animal protein and labor for carbohydrates and “luxury” items [38]. An archaeological study of central and western Europe shows that farmers of the Linearbandkeramik culture and hunter-gatherers may have coexisted by spatial exclusion: “People of the LBK settled in exactly those areas only marginally exploited by hunter-gatherers and not … with … more intense hunter-gatherers exploitation” [39]. A definitive study combines information from ancient DNA and dietary stable isotope data to show “persuasive evidence for the prolonged coexistence of genetically distinct hunter-gatherers and farming groups over the course of the Neolithic in Central Europe” [7]. Thus, various lines of evidence argue against the ready conversion of hunter-gatherers to farming. Moreover, Bowles [40] compares the productivity of foraging with early farming and concludes that, if hunter-gatherers converted to farming, they did not do so “because cultivation of crops was simply a better way to make a living.” The motivation for conversion may have been social competition, perhaps for marriage partners [41]. Prolonged coexistence provides opportunities for conversion, but also entails day-to-day competition unless there is active avoidance.

Fort [29] provides valuable estimates of the conversion rate expressed as “the average number of hunter-gatherers converted by each farmer per generation.” These estimates (e.g. 2.3 ~ 4.7) are calculated from ethnographic records of Christian missionaries in contact with a larger number of hunter-gatherers. Since only “successful” interactions appear to be included, the true conversion rate may be lower. In addition, it is not clear whether relations between missionaries and hunter-gatherers can be regarded as typical of what transpired in prehistoric encounters between farmers and hunter-gatherers.

There was apparently a considerable time delay between first contact of the indigenous hunter-gatherers with the incoming farmers and conversion of the former to farming; conversion may have occurred in the wake of the traveling wave rather than at the wave front. If true, there are at least two implications worth considering. First, it may be interesting to introduce into the model a time delay between contact and conversion, similar to the fixed time interval between migration events assumed by Fort and Méndez [28] and Fort [29]. Second, although conversion and intermarriage are not synonymous, if intermarriage also occurred well behind the wave front, then the “surfing” effect resulting in the introgression of European hunter-gatherer genes into the farming populations of Anatolian descent might have been considerably weakened [42] (see also Edmonds et al. [43]).

The model proposed in this paper predicts that the farmers of Anatolian descent will invade at below their intrinsic carrying capacity (i.e. the carrying capacity in the absence of competition with the converted farmers and hunter-gatherers, which equals unity in the non-dimensional variables; see Eq 5 and Fig 2) and, depending on the parameter values, well below it (Figs 3 and 4). With the exception of this difference and possibly a slower predicted speed (see Eq 7A), an equally good qualitative description of the wave-of-advance of the incoming farmers is provided by the original Fisher model [15]. Since a low conversion rate of hunter-gatherers to farming is required by the three-population model for it to apply, the motivation for invoking it in lieu of the simpler one-population Fisher model may be questioned. However, the speed of the spread of farming may in fact be slower [20, 21] than originally thought (~1 km/yr [11]), which in terms of the present model may perhaps be interpreted as due to the competitive effect, *w*, of hunter-gatherers on the alien farmers. In addition, if future empirical work can show that the first European farmers were well below their intrinsic carrying capacity because of competition with the hunter-gatherers, the present approach—or at the very least a two-population Lotka-Volterra competition model for the alien farmers and resident hunter-gatherers—may be justified.

Changes in the relative sizes of populations over time have been investigated using the method of summed probability distributions. This method assumes that the number of reported radiocarbon dates from all known sites in a given area and falling within a given time window can serve as an approximate proxy for the relative population size of that space-time sector [44, 45]. The summed probability distribution is a plot of these numbers over the period of interest. Applying this method to the Neolithic transition in Europe, Shennan et al. [30] identified a boom-bust temporal pattern at regional levels, which they tentatively attributed to endogenous causes such as population growth to unsustainable levels. On the other hand, an exponential growth curve apparently gives a good fit to the summed probability distribution for the whole of western Europe. Silva and Vander Linden [31] obtain similar results but interpret them differently.

The traveling wave solution pictured in Fig 2 suggests that the arrival of the wave-of-advance in any region entails exponential growth followed by demographic stasis of the incoming farmers. In other words, the summed probability distribution is expected to be flat after the initial exponential phase. It is not clear why a bust should follow the boom, but it may be as Shennan et al. [30] suggest that resources are depleted. Note this effect is not incorporated in the current wave-of-advance model, which does not address events subsequent to the initial spread of farming. At the pan-European level, the summed probability distribution should be monotone increasing as indeed it is, but I suggest that the null hypothesis for dependence on time should be closer to quadratic than exponential. This is because, given a constant radial rate of expansion in a planar habitat—as opposed to the linear habitat assumed in the present model—the total surface area occupied by the farmers should increase at a quadratic rate, whereas the density will be uniform throughout the occupied area.

## Appendix 1: Equilibria of the spatially homogeneous model

The non-dimensional equations ignoring spatial structure are

$$\begin{array}{l} F_{t}=aF(1-F-C-wH)\\ C_{t}=C(1-F-C-pH)+s(F+C)H\\ H_{t}=bH[1-H-(q+g)(F+C)] \end{array}$$ (A1)

If we ignore the term *sFH* in the second of these equations, Eq A1 reduces to a Lotka-Volterra three-population competition model. The equilibria of Eq A1 are:

$$E1.\;\hat{F}=0,\hat{C}=0,\hat{H}=0,$$

$$E2.\;\hat{F}=0,\hat{C}=0,\hat{H}=1,$$

$$E3.\;\hat{F}+\hat{C}=1,\hat{H}=0,$$

$$E4.\;\hat{F}=0,\hat{C}=\frac{1+s-p}{1+(s-p)(q+g)},\hat{H}=\frac{1-q-g}{1+(s-p)(q+g)},$$

$$E5.\;\hat{F}=\frac{\left(1-w)(p-w-s\right)}{\left(p-w)[1-w(q+g)\right]},\hat{C}=\frac{s(1-w)}{\left(p-w)[1-w(q+g)\right]},\hat{H}=\frac{1-q-g}{1-w(q+g)}.$$

The Jacobian is

$$\left(\begin{array}{ccc} a(1-2\hat{F}-\hat{C}-w\hat{H}( & -a\hat{F} & -aw\hat{F}\\ -\hat{C}+s\hat{H} & 1-\hat{F}-2\hat{C}+(s-p)\hat{H} & s\hat{F}+(s-p)\hat{C}\\ -b(q+g)\hat{H} & -b(q+g)\hat{H} & b\left[1-(q+g)(\hat{F}+\hat{C})-2\hat{H}\right] \end{array}\right)$$ (A2)

In reporting the results of local stability analysis, I will follow standard practice and write that an equilibrium is “linearly stable” if all (three) eigenvalues are negative real or have negative real parts, and “unstable” if at least one eigenvalue is positive real or has a positive real part. In the one case referred to as “neutrally stable”, one eigenvalue is zero and the other two are negative real. Equilibrium E1 is unstable. E2 is linearly stable if and only if (henceforth abbreviated as iff) *w* > 1 and *p* > 1 + *s*. E3 is neutrally stable if *q* + *g* > 1.

E4 exists iff 1+(*s*−*p*)(*q*+*g*), 1+*s*−*p*, and 1−*q*−*g* are all of the same sign. One eigenvalue at E4 is

$$\lambda_{1}=a\left[1-\frac{1+s-p+w(1-q-g)}{1+(s-p)(q+g)}\right].$$

The other two are the zeroes of the quadratic

$$f(\lambda )=\lambda^{2}+(\hat{C}+b\hat{H})\lambda +b\hat{C}\hat{H}[1+(s-p)(q+g)],$$

which given existence of this equilibrium are both negative or have negative real parts iff 1+(*s*−*p*)(*q*+*g*)>0. Hence, given 1+(*s*−*p*)(*q*+*g*)>0, *λ*_1_ is also negative iff (*p*−*w*−*s*)(1−*q*−*g*)<0. Thus, E4 exists and is linearly stable iff *p* < 1 + *s*, *q* + *g* < 1, and *p* < *w* + *s*.

## Appendix 2: Existence and linear stability of E5

The conditions for E5 to exist can be obtained as follows. First assume *w*(*q*+*g*)<1. Then $\hat{H}>0$ iff *q* + *g* < 1. Similarly, $\hat{C}>0$ iff either both *w* < 1 and *w* < *p* or both *w* > 1 and *w* > *p*, which entails $\hat{F}>0$ iff *p* > *w* + *s*. But *p* > *w* + *s* entails *w* < *p*. Note *q* + *g* < 1 and *w* < 1 imply *w*(*q*+*g*)<1. Next assume *w*(*q*+*g*)>1. Then $\hat{H}>0$ iff *q* + *g* > 1, and $\hat{C}>0$ iff either both *w* < 1 and *w* > *p* or both *w* > 1 and *w* < *p*, which entails $\hat{F}>0$ iff *p* > *w* + *s*. But again *p* > *w* + *s* entails *w* < *p*. Note *q* + *g* > 1 and *w* > 1 imply *w*(*q*+*g*)>1. Thus to summarize so far, E5 exists iff either

p>w+s,w<1,q+g<1 (A3A)

or

p>w+s,1<w<p,q+g>1 (A3B)

holds.

Given inequalities Eq A3A, the parameter dependence of the equilibrium values is as follows: $\hat{F}$ is monotone increasing in *p* and *q*+*g*, and monotone decreasing in *s* and *w*; $\hat{C}$ is monotone increasing in *s* and *q*+*g*, monotone increasing in *w* provided *p* < 1 (a maximum exists at $w=1-\sqrt{\left(1-p)[1-1/\left(q+g\right)\right]}$ if *p* >1), and monotone decreasing in *p*; $\hat{H}$ is monotone increasing in *w*, and monotone decreasing in *q*+*g*. Inequalities Eq A3B are inconsistent with linear stability as shown below.

Next, invoking Eq A2 and noting

$$\hat{F}+\hat{C}+w\hat{H}=1,$$

$$(q+g)(\hat{F}+\hat{C})+\hat{H}=1,$$

the characteristic polynomial at E5 is given by

$$f(\lambda )=\left|\begin{array}{ccc} -a\hat{F}-\lambda & -a\hat{F} & -aw\hat{F}\\ -\hat{C}+s\hat{H} & -\hat{C}-(p-w-s)\hat{H}-\lambda & s\hat{F}+(s-p)\hat{C}\\ -b(q+g)\hat{H} & -b(q+g)\hat{H} & -b\hat{H}-\lambda \end{array}\right|.$$

The cubic *f*(*λ*)→−∞ as *λ*→∞, and hence a positive or zero real eigenvalue exists if *f*(0) ≥ 0. Thus, in order for E5 to be linearly stable, we need

$$f(0)=ab(p-s-w)(w-1){\hat{H}}^{2}<0.$$

Recall that existence of E5 requires *p* > *w* + *s* (Eqs A3A and A3B). Hence, E5 given that it exists can only be linearly stable if *w* < 1. This entails that the conditions Eq A3A are sufficient for the existence of E5 and at the same time satisfy necessary conditions for its linear stability. On the other hand, E5 cannot be stable when the existence conditions Eq A3B hold.

We conclude from the above that neither E2, E3, nor E4 can coexist as linearly stable equilibria with E5. In particular, this rules out bistable traveling waves that connect E5 to E2.

## Appendix 3: Heuristic phase space analysis

Next we assume *F*, *C*, and *H* are functions of *x*−*vt* only, where *v* is the speed of the traveling wave, to conduct a heuristic phase space analysis.

$$\begin{array}{l} F^{\prime }=U\\ U^{\prime }=-aF(1-F-C-wH)-vU\\ C^{\prime }=V\\ V^{\prime }=-C(1-F-C-pH)-s(F+C)H-vV\\ H^{\prime }=W\\ W^{\prime }=-bH[1-H-(q+g)(F+C)]-vW \end{array}$$ (A4)

We investigate the eigenstructure of the equilibrium *E* = (0,0,0,0,1,0) for the case where E5 exists and necessary conditions for its linear stability are satisfied, i.e. when *p* > *w* + *s*, *w* < 1, *q* + *g* < 1 (Eq A3A). This entails that E2 is unstable. The characteristic polynomial is

$$\phi (\lambda )=\left|\begin{array}{cccccc} -\lambda & 1 & 0 & 0 & 0 & 0\\ -a(1-w) & -v-\lambda & 0 & 0 & 0 & 0\\ 0 & 0 & -\lambda & 1 & 0 & 0\\ -s & 0 & -(1-p+s) & -v-\lambda & 0 & 0\\ 0 & 0 & 0 & 0 & -\lambda & 1\\ b(q+g) & 0 & b(q+g) & 0 & b & -v-\lambda \end{array}\right|$$ (A5)

The eigenvalues that may be negative or have negative real part are

$$\lambda_{\pm }=\frac{-v\pm \sqrt{v^{2}-4a(1-w)}}{2}$$ (A6A)

$$\kappa_{\pm }=\frac{-v\pm \sqrt{v^{2}-4(1-p+s)}}{2}$$ (A6B)

$$\mu_{-}=\frac{-v-\sqrt{v^{2}+4b}}{2}$$ (A6C)

where *κ*_+_ requires 1 − *p* + *s* > 0. The corresponding stable eigenvectors are

$$\left(\begin{array}{l} a(1-w)-(1+s-p),[a(1-w)-(1+s-p)]\lambda_{\pm },s,s\lambda_{\pm },\\ \;-\frac{b(q+g)}{a(1-w)+b}[a(1-w)-1+p],-\frac{b(q+g)}{a(1-w)+b}[a(1-w)-1+p]\lambda_{\pm } \end{array}\right)$$ (A7A)

$$\left(0,0,1,\kappa_{\pm },-\frac{b(q+g)}{1+s-p+b},-\frac{b(q+g)}{1+s-p+b}\kappa_{\pm }\right)$$ (A7B)

$$\left(0,0,0,0,1,\mu_{-}\right)$$ (A7C)

The *F* and *C* components of the stable eigenvector cannot be of opposite signs. If *a*(1−*w*)>1+*s*−*p*, the subspace spanned by the eigenvectors A7a can serve as the direction of entry of a permissible orbit in phase space; the predicted minimum speed is then $v=2\sqrt{a(1-w)}$ from Eq A6A. If *a*(1−*w*)<1+*s*−*p* on the other hand, a similar argument using Eq A6B shows that $v=2\sqrt{1+s-p}$ may be the minimum speed.
